# Single-port robot-assisted radical prostatectomy

**DOI:** 10.1007/s00345-024-04914-5

**Published:** 2024-04-20

**Authors:** Nicolas A. Soputro, Jihad Kaouk

**Affiliations:** Glickman Urological & Kidney Institute, Cleveland Clinic, 9500 Euclid Avenue, Glickman Tower, Q10, Cleveland, OH 44195 USA

**Keywords:** Robotic surgery, Minimally invasive surgery, Single port, Radical prostatectomy, Prostate cancer

## Abstract

**Purpose:**

To provide a comprehensive update on the different techniques and outcomes of contemporary Single-Port (SP) Robotic Radical Prostatectomy (RARP) approaches.

**Methods:**

A literature review was performed to identify cohort studies that have utilized the purpose-built SP robotic platform (Intuitive Surgical Inc., Sunnyvale, California) for RARP. All published approaches of SP-RARP were included in our review. Baseline clinical, perioperative, and postoperative oncological and functional outcomes were collected from the included studies.

**Results:**

A total of 16 studies involving 1159 patients were identified. To date, five approaches of SP-RARP have been described, namely Transperitoneal, Extraperitoneal, Retzius-Sparing, Transperineal, and Transvesical. The surgical steps and clinical outcomes of the aforementioned approaches were discussed. While operating times were still faster in the Transperitoneal and Extraperitoneal cohorts, the novel and more regionalized Transvesical approach allowed for radical prostatectomy to be pursued in more patients with previous abdominal surgeries and contributed to significantly improved postoperative outcomes, including the earlier return of urinary continence and with most patients being discharged on the same day without any opioids.

**Conclusion:**

Based on the existing literature, the introduction of SP-RARP not only enriched the repertoire of minimally-invasive surgical treatment options for prostate cancer but also provided the opportunity for urologists to develop new techniques that can improve perioperative outcomes and postoperative quality of life. Given the limited number of patients and heterogeneity in the patient selection and reporting of postoperative outcomes, further research remains necessary to better understand the different benefits and improve patient selection algorithms for the different techniques.

**Supplementary Information:**

The online version contains supplementary material available at 10.1007/s00345-024-04914-5.

## Introduction

Since the introduction of robotic surgery in 1999, robotic radical prostatectomy (RARP) has been one of the mainstay treatments for localized clinically significant prostate cancer. The Food & Drug Administration (FDA) approval of the purpose-built Single-Port (SP) robotic platform (Intuitive Surgical Inc., Sunnyvale, California) in 2018 provided an addition to the repertoire of minimally-invasive surgical options for prostate cancer. When compared with the multiport (MP) platform, the SP system possessed several distinguishing features, such as a narrow profile and double-articulating instruments, which can facilitate multi-quadrant surgery from a single pivot point and offer improved maneuverability in a small surgical working space. These features provided a unique opportunity for surgeons to regionalize surgeries to the relevant anatomy, which can contribute significant benefits in enhancing perioperative outcomes, improving postoperative quality of life, and tailoring surgical approaches to the individual patient [1–3].

To date, different approaches of SP-RARP have been introduced, including transperitoneal, extraperitoneal, Retzius sparing, transperineal, and the more localized transvesical access (Supplementary Fig. 1A). With increasing variations in contemporary RARP practices, the objective of this review is to comprehensively analyze the existing literature on the differences in techniques and outcomes of currently available SP-RARP approaches.

## Methods

### Study design

A review of the literature was performed using Medline, PubMed, and Embase databases to identify all publications pertaining to SP-RARP between January 1st, 2020 and June 14th, 2023. The following search terms were used: “Single Port”, “Robotic Radical Prostatectomy”, and “Robot-Assisted Radical Prostatectomy”. All studies that involved a cohort of patients who underwent SP-RARP were included. Separate publications that utilized data from the same cohort were appropriately identified. Previous studies on Robotic Laparoendoscopic Single-Site surgery (R-LESS) and other studies that did not utilize the purpose-built SP robotic platform, review articles, animal studies, as well as articles not written in English were excluded. Data pertaining to the baseline clinical, perioperative, as well as postoperative oncological and functional outcomes were collected from the different studies.

### Description of technique

All SP-RARP cases were completed using the SP robotic platform. Following induction of anesthesia and patient positioning, all double-articulating instruments, camera, and the Remotely Operated Suction Irrigation (ROSI) system (Vascular Technology Inc. (VTI), Nashua, New Hampshire) were passed through the multichannel cannula of the purpose-built SP Access Kit (Intuitive Surgical Inc., Sunnyvale, California) that was inserted via a single 3.5 cm incision. The SP Access Kit possessed a separate channel that can accommodate an AirSeal trocar (ConMed, Utica, New York) for insufflation. The bubble port design of the SP Access Kit was particularly important to facilitate the “floating-dock” technique given the improved flexibility of the SP instruments that require at least 10cm of distance from the tip of the trocar to articulate [4]. The use of an additional assistant surgical port was based on the individual surgeon’s preference or specific intraoperative factors. Both transperitoneal and extraperitoneal SP-RARP can be performed with techniques similar to the corresponding MP approaches as previously described [1, 5–8].

The surgical technique for Transvesical SP-RARP was first described by Kaouk et al*.* [2], which involved supine patient positioning and direct access to the bladder via a midline suprapubic incision (Supplementary Fig. 1B). Following the insertion of the SP Access Kit, the bladder was insufflated up to a maximum of 12 mmHg and the SP robot was docked. Lower insufflation is generally favored in the Transvesical and Extraperitoneal techniques, especially given the higher risk of carbon dioxide absorption when compared to the Transperitoneal approach. Upon entry, the ureteral orifices were identified and the bladder neck was marked circumferentially with electrocautery. Dissection was commenced posteriorly and continued down to the vas deferens and seminal vesicles. After the posterior plane was developed, dissections were continued laterally on both sides to expose the endopelvic fascia. The dorsal venous complex (DVC) was then sutured and transected. For nerve-sparing procedures, newer techniques have been developed for complete sparing of the endopelvic fascia and DVC. Dissections were completed as the urethra was divided just distal to the apex of the prostate, aiming for maximal preservation of urethral length. Without undocking the robot, the intact specimen can be removed from the bladder and placed within the chamber of the bubble port. Starting with posterior reconstruction, vesicourethral anastomosis (VUA) was continued anteriorly in a clockwise and anticlockwise direction using two unidirectional barbed sutures (V-loc™, Covidien, Minneapolis, Minnesota). The purpose-built SP platform can facilitate the completion of VUA in the Transvesical approach entirely from within the confines of the bladder [2, 9].

**Fig. 1:**
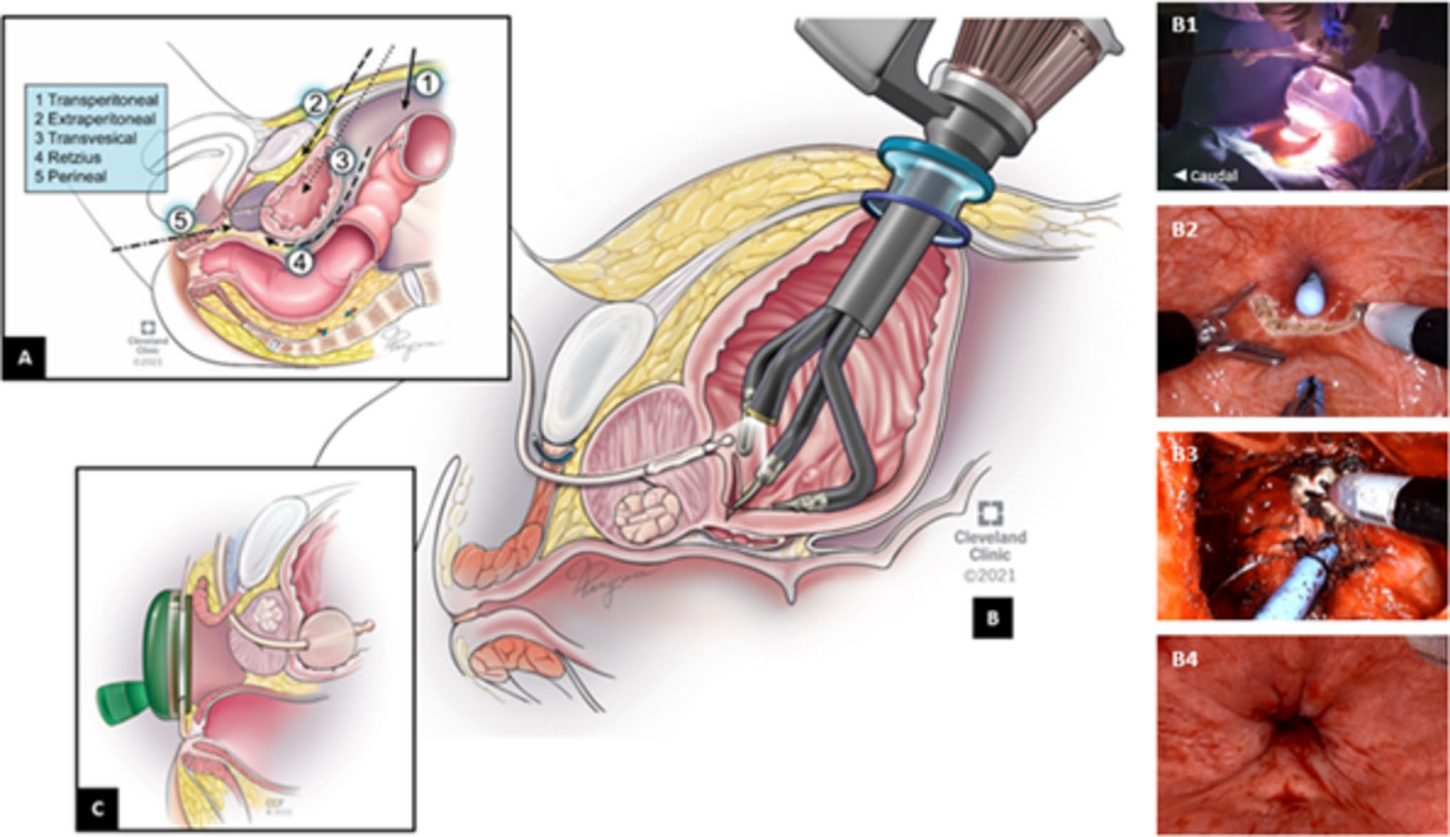
**A** Different approaches of Single-Port (SP) Robotic Radical Prostatectomy (RARP). **B** Transvesical SP-RARP with intraoperative images of the different steps, including **B1** placement of the SP Access Kit, **B2** commencement of posterior bladder neck dissection, **B3** apical dissection, and **B4** completion of the vesicourethral anastomosis; **C** transperineal SP-RARP

Transperineal SP-RARP was introduced by Lenfant et al*.* in 2021 (Supplementary Fig. 1C). With patients positioned in a dorsal lithotomy with 10° Trendelenburg, access to the perineum was obtained via a semilunar incision between the ischial tuberosities. Following docking of the SP robot, dissection was performed posteriorly to separate the levator ani muscles and to open the Denonvillier’s fascia. Dissections were then continued laterally to ligate the prostatic pedicles on both sides. Anteriorly, the prostatic apex and the urethra were transected first prior to dissecting the bladder neck. VUA was started anteriorly using barbed sutures and was completed in a running continuous fashion [10] (see Fig. 1).

## Results

### Pre-clinical and early clinical experience

The first series of SP-RARP was performed by Kaouk et al*.* in 2010 as part of a clinical trial using the Da Vinci SP999 (Intuitive Surgical Inc., Sunnyvale, California). The procedure was completed successfully in 11 patients without evidence of intraoperative complications or the need for conversion [11]. Following FDA approval, the first clinical experience of SP-RARP was published by Kaouk et al*.* in 2018. The operating times for the two patients were 140 min and intraoperative blood loss was minimal. There were no intraoperative complications and both patients were discharged home within 24 h [3].

### Transperitoneal SP-RARP

Baseline demographic and clinical characteristics were presented in Supplementary Table 1. Perioperative, oncological and functional outcomes were summarized in Table 1. Transperitoneal SP-RARP has been performed in at least 391 patients. The most common indication was intermediate-risk prostate cancer. Two institutions were noted to incorporate an additional 12 mm assistant trocar as part of their routine practice. LND was performed in 52.6% of patients. The operating times and estimated intraoperative blood loss (EBL) ranged between 150 and 250 min and 50 and 200 mL, respectively. Intraoperative complications were reported in three cases, one of which was a bowel injury during adhesiolysis. No surgical drain was placed at the end of the procedures. Most patients were discharged within 24 h and the rate of postoperative complications ranged between 2.5 and 26.1% [1, 5, 6, 12–15].

**Table 1:** Perioperative and postoperative outcomes of the previously published cohort studies on Single-Port (SP) Robotic Radical Prostatectomy (RARP)

	*n*	Operative time (min), median (IQR)	EBL (mL), median (IQR)	Extra port, *n* (%)	Lymph node dissection, *n* (%)	Surgical drain placement, *n* (%)	Intraoperative complications, *n* (%)	Length of stay (hours), median (IQR	Postoperative complication, *n* (%)
Transperitoneal									
Abou Zeinab et al*.* (2023) [1, 5, 15]	238	155 (110.5–249.8)	75 (50–150)	100 (42%)	101 (52.9%)	0 (0%)	2 (1.3%)	14 (13–24)	**Total** = 32 (13.4%) **Major** = 10 (4.2%)
Noh et al*.* (2022) [16]	31	Mean 151.3 ± 15.1	Mean 121.1 ± 64.7	31 (100%)	5 (16.1%)	0 (0%)	0 (0%)		**Clavien ≥ 2** = 0 (0%)
Balasubramanian et al*.* (2022) [6]	39	Mean 248 ± 36	Mean 130 ± 70		37 (94.9%)	0 (0%)	0 (0%)	*No. of days*: **1** = 37 (94.9%) **2** = 2 (5.1%)	**Clavien ≥ 2** = 2 (5.1%)
Kim et al*.* (2020) [14]	20	245 (200–255)	200 (150–300)	0 (0%)	11 (55%)	0 (0%)	0 (0%)	7 days (4–8)	**Clavien ≥ 2** = 0 (0%)
Jones et al*.* (2020) [13]	23	236 (191–343)	50 (20–500)	0 (0%)	12 (52.2%)	0 (0%)	1 (4.3%)	1 day (range, 1–6)	**Clavien ≥ 2** = 6 (26.1%)
Abaza et al*.* (2020) [12]	40	Mean 173.1 (147–216)	Mean 68.1 (25–250)	0 (0%)	40 (100%)	0 (0%)	0 (0%)	Mean 0.12 days (0–1 day)	**Total** = 1 (2.5%) **Major** = 0 (0%)
Extraperitoneal									
Abou Zeinab et al*.* (2023) [5]	238	206 (182.5–240)	150 (100–200)	0 (0%)	192 (84.6%)	2 (1.3%)	0 (0%)	7.5 (4.6–24.4)	**Total** = 39 (16.4%) **Major** = 18 (7.6%)
Kim et al*.* (2022) [7]	157	195 (165–221.25)	100 (100–200)			72 (45.9%)		*No. of days*: **0** = 110 (70%) **1** = 45 (29%) **2** = 2 (1%)	**Total** = 6 (3.8%) **Major** = 4 (2.5%)
Balasubramanian et al*.* (2022)[6]	30	Mean 224 ± 41	Mean 138 ± 87		23 (76.7%)	1 (3.3%)	2 (6.7%)	7	**Clavien ≥ 2** = 3 (10%)
Agarwal et al*.* (2020)[18]	49	161 (134–194)	200 (75–300)	0 (0%)	38 (78%)	1 (2%)	0 (0%)	7 (7–8)	**Total** = 4 (8%) **Major** = 0 (0%)
Retzius-Sparing									
Bassett et al*.* (2022) [19]	28	Mean 234 ± 61	Mean 148 ± 102	0 (0%)	24 (86%)	0 (0%)	0 (0%)		**Total** = 4 (14%) **Major** = 2 (7%)
Koukourikis et al*.* (2022) [8]	10	106 (101–109)	125 (50–150)	0 (0%)		0 (0%)	0 (0%)	8 (7–9)	**Total** = 1 (10%) **Major** = 0 (0%)
Balasubramanian et al*.* (2022) [6]	32	Mean 208 ± 40	Mean 112 ± 46		30 (93.8%)	0 (0%)	0 (0%)	7	**Clavien ≥ 2** = 5 (15.6%)
Transperineal									
Lenfant et al*.* (2021) [10]	26	255 (204–289)	100 (50–150)	0 (0%)	16 (61.5%)	1 (4.8%)		11 (8–23)	**Total** = 13 (50%) **Major** = 6 (23%)
Transvesical									
Ramos et al*.* (2023) [2, 9]	100	212.5 (188–238.8)	100 (50–150)	0 (0%)	43 (43%)	0 (0%)	0 (0%)	3 (3–4)	**Total** = 16 (16%) **Major** = 2 (2%)

	Pathology Gleason scores, *n* (%)	Pathology T stage, *n* (%)	Positive margins, *n* (%)	Biochemical recurrence, *n* (%)	Foley Catheter Duration (days), median (IQR)	Continence, *n* (%)	Potency, *n* (%)
Transperitoneal							
Abou Zeinab et al*.* (2023) [1, 5, 15]		**pT2** = 107 (72.3%) **pT3a** = 31 (20.9%) **pT3b** = 7 (4.7%) **pT4** = 3 (2%)	61 (26.9%)		5 (4–5.2)	**6 weeks** = 68 (40.2%) **3 months** = 133 (63%) **6 months** = 163 (87.2%)	
Noh et al*.* (2022) [16]	**Group 1** = 4 (12.9%) **Group 2** = 14 (45.1%) **Group 3** = 10 (32.2%) **Group 4** = 2 (6.5%) **Group 5** = 1 (3.2%)	**pT2** = 22 (71%) ** ≥ pT3a** = 9 (29%)	6 (19.4%)	0 (0%)	7	**2 weeks** = 48.4% **1 month** = 67.7% **3 months** = 77.4% **6 months** = 80.6%	**2 weeks** = 19.4% **1 month** = 22.6% **3 months** = 45.2% **6 months** = 67.7%
Balasubramanian et al*.* (2022) [6]	**Group 1** = 4 (10.3%) **Group 2** = 20 (51.3%) **Group 3** = 9 (23.1%) **Group 4** = 3 (7.7%) **Group 5** = 3 (7.7%)	**pT2** = 21 (53.8%) **pT3a** = 12 (30.8%) **pT3b** = 6 (15.4%)	10 (25.6%)	6 (15.4%)	7	**Time to continence (days)**: mean 116 ± 85.1	**Time to erection (days)**: mean 134 ± 89.1
Kim et al*.* (2020) [14]	**Group 1** = 1 (5%) **Group 2** = 7 (35%) **Group 3** = 3 (15%) **Group 4** = 3 (15%) **Group 5** = 6 (30%)	**pT2** = 11 (55%) **pT3a** = 5 (25%) **pT3b** = 3 (15%) **pT4** = 1 (5%)	7 (35%)	**3 months** = 1 (10%)	8	**3 months** = 10 (100%)	**3 months** = 4 (57.1%)
Jones et al*.* (2020) [13]			9 (23.1%)	*Detectable PSA*: 3 (13%)	9 (range, 6–34)		
Abaza et al*.* (2020) [12]	**Gleason 6** = 2 (5%) **Gleason 7** = 31 (80%) **Gleason ≥ 8** = 6 (15%)	**pT2** = 18 (45%) **pT3a** = 14 (35%) **pT3b** = 6 (15%)	9 (23%)				
Extraperitoneal							
Abou Zeinab et al*.* (2023) [5]		**pT2** = 111 (61.7%) **pT3a** = 50 (27.8%) **pT3b** = 14 (7.8%) **pT4** = 5 (2.8%)	55 (23.3%)		7 (7–10)	**6 weeks** = 68 (40.2%) **3 months** = 133 (63%) **6 months** = 163 (87.2%)	
Kim et al*.* (2022) [7]			**Total** = 28% **pT2** = 26% **pT3a** = 36% **pT3b** = 40%	**9 months** = 8.3%		**9 months** = 82.5%	**9 months** = 64.4%
Balasubramanian et al*.* (2022)[6]	**Group 1** = 2 (6.7%) **Group 2** = 15 (50%) **Group 3** = 6 (20%) **Group 4** = 3 (10%) **Group 5** = 4 (13.3%)	**pT2** = 16 (53.3%) **pT3a** = 8 (26.7%) **pT3b** = 6 (20%)	8 (26.7%)	1 (3.7%)	7	**Time to continence (days)**: mean 84 ± 53.1	**Time to erection (days)**: mean 105 ± 99.3
Agarwal et al*.* (2020)[18]	**Gleason 6** = 4 (9%) **Gleason 7** = 37 (78%) **Gleason ≥ 8** = 6 (13%)	**pT2** = 40 (85%) **pT3a** = 4 (9%) **pT3b** = 3 (6%)	13 (28%)	*Detectable PSA*: **3 months** = 1 (3.6%)	7 (7–8)	**3 months** = 16 (76%)	**3 months** = 10 (48%)
Retzius-Sparing							
Bassett et al*.* (2022) [19]	**Group 2** = 16 (57%) **Group 3** = 8 (29%) **Group ≥ 4** = 4 (14%)	**pT2** = 15 (54%) **pT3a** = 11 (39%) **pT3b** = 1 (4%)	5 (18%)	**3 months** = 1 (3.6%)		**Immediate** = 23 (82%) **3 months** = 25 (89%) **6 months** = 27 (96%)	*Nerve sparing*: Postop SHIM, mean 18 ± 7 (Preop SHIM, mean 20 ± 6)
Koukourikis et al*.* (2022) [8]	**Group 1** = 0 (0%) **Group 2** = 5 (50%) **Group 3** = 4 (40%) **Group 4** = 1 (10%) **Group 5** = 0 (0%)	**pT2** = 6 (60%) **pT3a** = 3 (30%) **pT3b** = 1 (10%)	5 (50%)	**3 months** = 0 (0%)	8 (7–9)	**3 months** = 10 (100%)	
Balasubramanian et al*.* (2022) [6]	**Group 1** = 1 (3.1%) **Group 2** = 20 (62.5%) **Group 3** = 6 (18.8%) **Group 4** = 2 (6.3%) **Group 5** = 3 (9.4%)	**pT2** = 28 (87.5%) **pT3a** = 3 (9.4%) **pT3b** = 1 (3.1%)	10 (31.3%)	6 (19.4%)	7	**Time to continence (days)**: mean 47.7 ± 37.2	**Time to erection (days)**: mean 47.2 ± 29.9
Transperineal							
Lenfant et al*.* (2021) [10]	**Group 1** = 1 (3.8%) **Group 2** = 14 (53.8%) **Group 3** = 9 (34.6%) **Group 4** = 2 (7.7%) **Group 5** = 0 (0%)	**pT2** = 14 (53.8%) **pT3a** = 9 (34.6%) **pT3b** = 3 (11.5%)	17 (65.4%)	**12 months** = 1 (3.8%)	11 (8–23)	**3 months** = 10 (52.6%) **6 months** = 15 (75%) **12 months** = 16 (80%)	
Transvesical							
Ramos et al*.* (2023) [2, 9]			15 (15%)	1 (1%)	3 (3–4)	**Immediate** = 49% **6 weeks** = 77.4% **3 months** = 86.8% **6 months** = 94.1% **12 months** = 98.9%	**SHIM > 10 at 6 months** = 17 (37%)

Positive surgical margin (PSM) was identified in up to 26.9%. A breakdown of the pathology Gleason scores and T stages were summarized in Table 1. The median Foley catheter duration of the group ranged between 5 and 9 days. Following Foley catheter removal, varying continence outcomes have been reported with a mean time to continence of 116 ± 85.1 days as highlighted by Balasubramanian et al*.* and continence rates of 40.2% and 87.2% at 6 weeks and at 6 months, respectively, as demonstrated in a multi-institutional study by Abou Zeinab et al. Furthermore, Balasubramanian et al. estimated the time to erection of 134 ± 89.1 days, which appeared to correlate with the potency rates reported by Noh et al. and Kim et al. [5, 6, 14, 16].

### Extraperitoneal & retzius-sparing SP-RARP

Extraperitoneal and Retzius-sparing SP-RARP were performed in 565 and 77 patients, respectively. The time taken to complete the two procedures ranged between 100 – 240 min, with EBL ranging between 50 and 200 mL. One institution reported routine use of an 8 mm assistant port in 38% of their extraperitoneal cohort [17]. Compared with the transperitoneal cohort, LND was more commonly pursued in 77–94% of patients who underwent extraperitoneal and Retzius-sparing SP-RARP. A surgical drain was placed in 75 patients, all of which following extraperitoneal SP-RARP. There were no intraoperative complications in the Retzius-sparing cohort. Of the four complications reported during the extraperitoneal approach, two were attributed to iatrogenic pneumothorices, which necessitated intraoperative placement of intercostal catheters [6, 17]. Similar to transperitoneal SP-RARP, most patients were discharged within 24 h with a relatively low risk of major postoperative complications (0–15%) [5–8, 18, 19].

The PSM rates of 17–28% in the extraperitoneal cohort appeared to be better compared to the PSM rates of up to 50% in the Retzius-sparing series. Of note, one of the institutions that contributed to the multi-institutional extraperitoneal SP-RARP series by Abou Zeinab et al*.* identified that 50% of their PSMs were focal involvements [20]. When analyzed based on the different pathology T stages, Kim et al*.* identified the PSM rates of 26%, 36%, and 40% for pT2, pT3a, and pT3b, respectively [7]. Nevertheless, the biochemical recurrence rates in most cases remained relatively low, as summarized in Table 1 [6–8, 17, 19, 20].

The median Foley catheter duration was 7 days following the two approaches. When comparing between the Retzius-sparing and Extraperitoneal approaches, Balasubramanian et al*.* reported the mean times to continence of 47.7 ± 37.2 days and 84 ± 53.1 days following the two approaches, respectively [6]. Furthermore, despite the small cohort of 28 patients, Bassett et al*.* reported immediate continence in 82% after Retzius-sparing SP-RARP [19]. Similar to previous studies on transperitoneal SP-RARP, notable heterogeneity in the reporting of postoperative erectile functions can be appreciated for the extraperitoneal and Retzius-sparing series. While the multi-institutional article by Abou Zeinab et al*.* did not comment on sexual potency [5], several institutions that contributed to the cohort have reported on these outcomes separately. For example, Harrison et al*.* reported full erection in 37% and partial erection in 25% after 12 months, while Lenfant et al*.* noted varying degrees of erectile function in 80% after 12 months [17, 20].

### Transperineal SP-RARP

The introduction of Transperineal SP-RARP provided an alternative in whom conventional robotic surgical treatments may be challenging or contraindicated, especially in patients with previous abdominal surgery. When compared with the transperitoneal MP-RARP cohort, previous history of laparotomy was identified to be more common in the 26 Transperineal patients (46% vs. 7.7%, *p* < 0.05). Perioperatively, although the Transperineal approach was associated with a reduced EBL (100 vs. 200 mL, *p* < 0.05), the median operating time was noted to be longer (255 vs. 163 min,* p* < 0.05). Postoperatively, however, the Transperineal approach contributed to a shorter length of inpatient stay (23 vs. 27, *p* < 0.05) and reduced opioid prescription on discharge (5 vs. 24,* p* < 0.05). Major postoperative complications were reported in six patients, which included three anastomotic leakages, two vesicourethral strictures, and one lymphocele requiring percutaneous drainage [10].

In terms of oncological outcomes, despite the higher prevalence of PSM in the Transperineal cohort (65.4% vs. 23%, *p* < 0.05), the biochemical recurrence rate at one year remained comparable between the two approaches. Foley catheter duration was longer following Transperineal SP-RARP (11 vs. 9 days, *p* < 0.05). Continence at 3, 6, and 12 months was achieved in 53%, 75%, and 80% of patients, respectively, which was similar to the continence outcomes for the transperitoneal MP-RARP cohort [10].

### Transvesical SP-RARP

The Transvesical approach represents the latest addition to the different armamentariums of SP-RARP. Similar to the Transperineal approach, many of the patients who underwent Transvesical SP-RARP had a previous history of abdominal surgery (49%). The median prostate volume was 33 mL and the most common indication was intermediate-risk prostate cancer (68.4%). All procedures were completed successfully without any intraoperative complications, need for conversion, or additional ports. The median operating time and EBL were 212 min and 100 mL, respectively. Limited LND was performed in 43%. When compared with other approaches of SP-RARP, the Transvesical cohort was noted to have the shortest length of inpatient stay with a median of 5.6 h and with 92% being discharged within 24 h. Major complications were reported in two patients with both being lymphoceles [2, 9].

In addition, the median Foley catheter duration of 3 days following Transvesical SP-RARP was significantly shorter compared to other approaches. Despite this, urinary retention was only identified in nine patients and there was no evidence of postoperative urine leak. Of note, the Transvesical approach was also associated with an earlier return of urinary continence with 49% reporting immediate continence following Foley removal and 87% reporting the use of zero or security pad at 3 months. As presented in Table 1, 37% had a Sexual Health Inventory for Men (SHIM) score > 10 at 6 months postoperatively [2, 9].

## Discussion

In addition to expanding the minimally-invasive surgical treatment options for prostate cancer, the purpose-built SP robotic platform has paved the way for the regionalization of RARP with novel techniques, such as with the SP Transvesical approach. Given the opportunity to customize our surgical approach to the individual patient, appreciating the differences in the perioperative, oncological, and functional outcomes of the various SP RARP approaches have become increasingly important. This literature review was the first, to our knowledge, to comprehensively describe the different techniques and outcomes of the SP-RARP approaches that have been utilized thus far, namely Transperitoneal, Extraperitoneal, Retzius-sparing, Transperineal, and Transvesical SP-RARP.

Previous studies have demonstrated that the SP platform can be safely and effectively used to perform conventional transperitoneal and extraperitoneal RARP with minimal modifications to the respective gold-standard MP-RARP techniques and with relatively low rates of perioperative complications (Table 1) [5–7, 12–14, 18]. A recent analysis comparing MP and SP Transperitoneal RARP by Noh et al*.* identified similar intraoperative console times, EBL, PSM rates, as well as 3-month continence and potency rates between the two robotic systems [16]. A different analysis comparing Extraperitoneal SP-RARP and Transperitoneal MP-RARP by Lenfant et al*.* also reported identical intraoperative outcomes between the two techniques, but with a significantly reduced length of stay (4.3 vs. 26.1 h, *p* < 0.05) and postoperative opioid prescriptions, both as an inpatient (32% vs. 64%, *p* < 0.05) and on discharge (35% vs. 87%, *p* < 0.05) in the SP cohort [20]. A later series by Harrison et al*.* echoed similar findings, highlighting the enhanced postoperative outcomes following Extraperitoneal SP-RARP [17].

Evidence of enhanced postoperative recovery was more apparent following the more localized Transvesical approach. In addition to the reduced length of stay, opioid use, and Foley catheter duration, Transvesical SP-RARP was also associated with an earlier return of continence. This may be attributed to the maximal preservation of the urethral stump length and the minimal disruption to the supporting structures of the bladder that can be achieved with the novel approach, which represents an extension to the principle often adopted in Retzius-Sparing RARP [2, 9]. Recently, other benefits of Transvesical SP-RARP have also been demonstrated, including its role as an alternative in patients with morbid obesity and previous abdominal surgeries, as well as to facilitate the development of surgical focal therapy options in the form of partial prostatectomy [21]. Furthermore, with direct percutaneous entry into the bladder, Transvesical SP-RARP no longer required the steep Trendelenburg position that was necessary in other conventional RARP techniques. The supine positioning may also apply to other SP-RARP approaches, which can provide the additional opportunity to perform RARP under regional anesthesia [22].

The opportunity to regionalize radical prostatectomy to the relevant anatomy, as facilitated by the SP platform, also translated to differences in the postoperative complications following various approaches of SP-RARP. Despite the limited number of studies and patients to date, the more localized Transvesical approach appeared to have maintained a relatively low risk of intraoperative bleeding and without any evidence of intraoperative complications [5–7, 12–14, 18]. When comparing the postoperative complications with other approaches, the Transvesical approach was also associated with reduced risks of non-urological complications, such as pneumothorax, ileus, and ventral hernia. The latter can be achieved given the complete preservation of the peritoneum albeit the lower incision [23, 24]. At our institution, we have been able to safely encourage patients to return to normal activities without any weight restrictions two weeks following their respective surgery [24]. Of note, the previous literature has identified the relatively higher incidence of symptomatic lymphocele of up to 5.8% following Extraperitoneal SP-RARP [5–7, 18]. Given the likely aetiology to be associated with postoperative fluid entrapment in the extraperitoneal space, various centers have adopted a technical modification which incorporated a small peritonotomy prior to fascial closure at the end of each procedure that resulted in a significantly reduction in symptomatic lymphocele.

It should be appreciated that the perioperative, oncological and functional outcomes included in Table 1 also reflected the different surgeons’ experiences early in the learning curve of using the SP platform. Despite the paucity of evidence surrounding the learning curve of the varying SP-RARP approaches, a recent study on the first 100 cases of Transvesical SP-RARP performed by a single surgeon identified that plateau performance was reached after the 28th case with a total console and operating times of 135 and 213 min, respectively [23]. These improvements in the learning curve and intraoperative experiences with the SP platform may contribute to the expanded surgical indications of the Transvesical approach, especially in patients with prostate volume greater than 80 mL at our institution, for example, Transvesical SP-RARP has been increasingly adopted in patients with larger glands of up to 150 mL.

This review was not devoid of limitations. The first relates to the descriptive nature of this literature review, in which the primary objective was to provide a comprehensive explanation of the techniques and clinical outcomes of the different SP-RARP approaches. Given the relative novelty of the platform and some of the approaches, the postoperative follow-up durations and the number of patients included in some of the studies remained limited. Several studies also employed different patient selection criteria, such as the Transvesical cohort that initially excluded patients with larger prostates and higher risk of lymph node involvement, which may introduce selection bias when attempting to compare the different SP-RARP approaches. Furthermore, there were notable heterogeneities and incomplete information pertaining to the reported outcomes in the different studies, especially on the postoperative functional outcomes. As such, there remains a limited amount of information to draw meaningful conclusions on the performance of certain SP-RARP approaches to other SP or MP techniques.

## Conclusion

In conclusion, the existing literature has demonstrated the safe and effective use of various SP-RARP approaches with favorable clinical outcomes and low perioperative morbidity. The purpose-built SP robotic platform allowed for the regionalization of RARP, such as with the Transvesical approach, which provided substantial benefits in enhancing perioperative outcomes and improving postoperative quality of life. With the increasing use of SP-RARP and the emergence of various approaches, it is imperative to appreciate the heterogeneity of the patient profiles and practices of the different surgeons. Hence, in conjunction with refining operating techniques in pursuit of better perioperative and long-term oncological and follow-up outcomes, future research can be directed towards prospective, multi-center studies to further improve our patient selection algorithm and to better understand the roles of SP-RARP within the contemporary armamentarium of RARP approaches.

## Supplementary Information

Below is the link to the electronic supplementary material.

Supplementary file1 (DOCX 28 KB)

## Data Availability

Data availability statement is not relevant given the nature of this manuscript as a literature review.
