# Supplementary material for: Single-port robot-assisted radical prostatectomy
Source: World J Urol. 2024 Apr 20;42(1):245. doi: 10.1007/s00345-024-04914-5 (PMC11032265; doi:10.1007/s00345-024-04914-5)
Supplement: Supplementary file 1 — Supplementary file1 (DOCX 28 KB) [file 345_2024_4914_MOESM1_ESM.docx]

**Supplementary Table 1**. Baseline clinical variables of the previously published studies on SP-RARP

|  | ***n*** | **Age (years), median (IQR)** | **BMI, (kg/m^2^), median (IQR)** | **Previous abdominal surgery, *n* (%)** | **PSA (ng/mL), median (IQR)** | **Prostate volume (mL), median (IQR)** | **Biopsy Pathology, *n* (%)** | **Risk Classifications, *n* (%)** |
| --- | --- | --- | --- | --- | --- | --- | --- | --- |
| **Transperitoneal** | | | | | | | | |
| Abou Zeinab *et al.* (2023)[1, 5, 15] | 238 | 63  (59 – 69) | 27  (25 – 30) | 1 (0.6%) | 6.5  (5.8 – 7) | 49  (44 – 61) | **Gleason 6** = 39 (16.4%)  **Gleason 7** = 183 (76.9%)  **Gleason 8** = 12 (5%)  **Gleason 9** = 3 (1.3%)  **Gleason 10** = 1 (0.4%) | *NCCN*:  **Low** = 38 (16%)  **Intermediate** = 181 (76.1%)  **High** = 19 (8%) |
| Noh *et al.* (2022)[16] | 31 | Mean 68.5 ± 6.3 | Mean 24.6 ± 2.8 |  | Mean 9.9 ± 6.2 | Mean 33.9 ± 10.8 | **Group 1** = 8 (25.8%)  **Group 2** = 12 (38.7%)  **Group 3** = 9 (29%)  **Group 4** = 2 (6.5%) |  |
| Balasubramanian *et al.* (2022)[6] | 39 | Mean 62.7 ± 6.8 | Mean 28.8 ± 4.3 | 6 (15.4%) | Mean 7.4 ± 5 |  | **Group 1** = 4 (10.3%)  **Group 2** = 23 (59%)  **Group 3** = 6 (15.4%)  **Group 4** = 5 (12.8%)  **Group 5** = 1 (2.6%) |  |
| Kim *et al.* (2020)[14] | 20 | 66  (60 – 71) | 24  (22.4 – 25.7) |  | 8.01  (5. 38 – 10.3) | 32  (25 – 45.4) |  | **Low** = 3 (15%)  **Intermediate** = 9 (45%)  **High** = 8 (40%) |
| Jones *et al.* (2020)[13] | 23 | 62  (Range, 48 – 77) | 30  (Range, 24.4 – 47.4) |  | 8.3  (Range, 6 – 40.7) | 48.6  (Range, 32 – 184) | **Gleason 6** = 3  **Gleason 7** = 15  **Gleason 8** = 3  **Gleason 9** = 1  **Gleason 10** = 1 |  |
| Abaza *et al.* (2020)[12] | 40 | Mean 64.2 ± 7 | Mean 25.4 ± 3.2 |  | Mean 7.9 ± 6.6 |  | **Gleason 6** = 7 (18%)  **Gleason 7** = 24 (60%)  **Gleason ≥8** = 9 (22%) |  |
| **Extraperitoneal** | | | | | | | | |
| Abou Zeinab *et al.* (2023)[5] | 238 | 64  (60 – 68) | 27  (25 – 30) | 49 (28.5%) | 6.5  (4.9 – 9) | 49  (42 – 62) | **Gleason 6** = 47 (19.7%)  **Gleason 7** = 178 (74.8%)  **Gleason 8** = 10 (4.2%)  **Gleason 9** = 3 (1.3%) | *NCCN*:  **Low** = 38 (16%)  **Intermediate** = 181 (76.1%)  **High** = 19 (8%) |
| Kim *et al.* (2022)[7] | 157 | 63  (59 – 68) | 27.8  (25.8 – 29.6) |  | 6.3  (4.7 – 8.2) |  | **Group 1** = 39 (24.8%)  **Group 2** = 83 (52.9%)  **Group 3** = 13 (8.3%)  **Group ≥4** = 22 (14%) |  |
| Harrison *et al.* (2022)[17] | 98 | 61.9  (57.4 – 65.6) | 27.1  (24.7 – 29.3) | 20 (20.4%) | 5.6  (4.4 – 8.1) | 37  (25.1 – 49.5) | **Group 1** = 24 (24.5%)  **Group 2** = 55 (56.1%)  **Group 3** = 13 (13.3%)  **Group 4** = 5 (5.1%)  **Group 5** = 1 (1%) | *D’Amico*:  **Low** = 24 (24.5%)  **Intermediate** = 67 (68.4%)  **High** = 7 (7.1%) |
| Balasubramanian *et al.* (2022)[6] | 30 | Mean 64.6 ± 8.6 | Mean 32.1 ± 6.4 | 6 (20%) | Mean 9.1 ± 5.5 |  | **Group 1** = 1 (3.3%)  **Group 2** = 14 (46.7%)  **Group 3** = 7 (23.3%)  **Group 4** = 3 (10%)  **Group 5** = 5 (16.7%) |  |
| Agarwal *et al.* (2020)[18] | 49* | 62  (58 – 66) | 28.5  (25.5 – 31.7) | 22 (45%) | 6.4  (4.9 – 9.1) |  |  |  |
| **Retzius-Sparing** | | | | | | | | |
| Bassett *et al.* (2022)[19] | 28 | Mean 65 ± 7 | Mean 25 ± 4 | 7 (25%) | Mean 10.2 ± 10.7 |  | **Group 1** = 2 (7%)  **Group 2** = 16 (57%)  **Group 3** = 5 (18%)  **Group ≥4** = 5 (18%) | *D’Amico*:  **Low** = 2 (7%)  **Intermediate** = 20 (71%)  **High** = 6 (21%) |
| Koukourikis *et al.* (2022)[8] | 10 | 70  (62.5 – 72) | 23.5 (21.3 – 24.3) |  | 7.4  (6.2 – 9.1) | 25.2  (20 – 33.3) | **Group 1** = 3 (30%)  **Group 2** = 4 (40%)  **Group 3** = 5 (50%) |  |
| Balasubramanian *et al.* (2022)[6] | 32 | Mean 62 ± 7.7 | Mean 28.7 ± 3.8 | 4 (12.5%) | Mean 7.1 ± 5.5 |  | **Group 1** = 2 (6.3%)  **Group 2** = 17 (53.1%)  **Group 3** = 8 (25%)  **Group 4** = 1 (3.1%)  **Group 5** = 3 (9.4%) |  |
| **Transperineal** | | | | | | | | |
| Lenfant *et al.* (2021)[10] | 26 | 64.1  (59.5 – 67.1) | 28.3  (26.4 – 32.3) | 22 (84.6%) | 6.5  (5.5 – 9.1) | 30  (24.9 – 41) | **Group 1** = 6 (23.1%)  **Group 2** = 12 (46.2%)  **Group 3** = 6 (23.1%)  **Group 4** = 2 (7.7%) | *NCCN*:  **Low** = 4 (16.7%)  **Intermediate** = 17 (70.8%)  **High** = 3 (12.5%) |
| **Transvesical** | | | | | | | | |
| Ramos *et al.* (2023)[2, 9] | 100 | 62.1  (58.1 – 66.3) | 28.1  (25.2 – 31.3) | 49 (49%) | 5  (4 – 8) | 33  (25 – 43.2) | **Group 1** = 29 (29%)  **Group 2** = 48 (48%)  **Group 3** = 18 (18%)  **Group 4** = 3 (3%)  **Group 5** = 2 (2%) | *NCCN*:  **Very Low** = 1 (1%) **Low** = 25 (25%)  **Intermediate** = 69 (69%)  **High** = 2 (2%)  **Very High** = 3 (3%) |
| IQR = Interquartile Range; BMI = Body Mass Index; PSA = Prostate Specific Antigen; NCCN = National Comprehensive Cancer Network | | | | | | | | |
